# Anti-reflux barrier competency can be estimated by gastric folds stretching during intragastric insufflation without special equipment

**DOI:** 10.1055/a-2697-7690

**Published:** 2025-09-24

**Authors:** Hidenori Tanaka, Haruhiro Inoue, Yuto Shimamura, Masachika Saino, Kei Ushikubo, Miyuki Iwasaki, Kazuki Yamamoto, Yohei Nishikawa, Ippei Tanaka, Mayo Tanabe, Satoshi Abiko, Gantuya Boldbaatar, Manabu Onimaru, Shiro Oka

**Affiliations:** 1 68272 Gastroenterology, Hiroshima University Hospital, Hiroshima, Japan; 2 378609 Digestive Diseases Center, Showa Medical University Koto Toyosu Hospital, Koto, Japan

**Keywords:** Endoscopy Upper GI Tract, Reflux disease, Motility / achalasia, Diagnosis and imaging (inc. chromoendoscopy, NBI, iSCAN, FICE, CLE)

## Abstract

**Background and study aims:**

Although the endoscopic pressure study integrated system (EPSIS) is useful to evaluate competency of lower esophageal sphincter as a major part of the anti-reflux barrier, its availability is limited. This study aimed to assess whether gastric fold stretching during insufflation can predict intragastric pressure (IGP) without special equipment.

**Patients and methods:**

A retrospective analysis included 33 patients who underwent esophagogastroduodenoscopy and EPSIS between June and July 2024. Gastric fold stretching along the greater curvature at the level of the cardia, observed in a retroflex view during insufflation, was compared with EPSIS results by reviewing recorded videos. Time ranges were defined as follows, and IGP was measured at the end of each range: Time range 1, until the ratio of longitudinal fold thickness to the groove width between folds reached 1:2; Time range 2, until the ratio reached 1:4; and Time range 3, until the folds or mucosal ridges were almost flattened. Variability was assessed using the coefficient of variation (CV), calculated as the standard deviation divided by the mean.

**Results:**

Time ranges 1, 2, and 3 were fully observed in 100%, 97%, and 70% of patients, respectively. Mean IGPs at the end of Time ranges 1, 2, and 3 were 8.9, 11.1, and 17.7 mmHg, with CVs of 0.32, 0.28, and 0.08, respectively.

**Conclusions:**

Flattening of gastric folds or mucosal ridges during insufflation is a reliable predictor of IGP. This finding may help identify patients with anti-reflux barrier dysfunction during regular endoscopic examination.

## Introduction

The endoscopic pressure study integrated system (EPSIS) is a tool designed to measure intragastric pressure (IGP) during CO _2_ insufflation in esophagogastroduodenoscopy (EGD) 1 2 3 4 5 6 . Previous studies have demonstrated that a maximum IGP below 18.7 mmHg and a flat IGP waveform pattern serve as predictors of gastroesophageal reflux disease (GERD) and Barrett’s esophagus 1 2 . In addition, objective parameters such as the pressure difference between basal and maximum IGPs and the gradient of the waveform have been identified as useful indicators for predicting abnormal acid reflux 3 . EPSIS has also proven valuable in diagnosing achalasia 4 . Despite its utility, EPSIS is not commercially available, remains in an early phase of dissemination, and requires an external measuring device connected to the endoscope via the working channel 7 . These circumstances have limited its routine use and the number of publications from other groups. Therefore, a practical, equipment-free surrogate that can be applied during standard EGD would be valuable in broader clinical settings. Specifically, the relationship between IGP and gastric distention during insufflation may offer a practical approach. As the stomach distends, flattening of the gastric folds may reflect the buffering of increased IGP, potentially allowing for IGP prediction based on these visual changes. The aim of this study was to evaluate whether dynamic endoscopic observations of gastric distention during insufflation can predict IGP.

## Patients and methods

### Patients

This retrospective study was conducted at Showa Medical University Koto Toyosu Hospital, including patients who underwent EGD and EPSIS procedures between June and July 2024. Retrospectively recorded video clips displaying dynamic endoscopic images and EPSIS-derived IGP curves side-by-side on a single split-screen were independently reviewed by two observers (HT and MS) to evaluate the relationship between gastric distention and IGP. For the main analysis, values were adopted based on mutual consensus between the two observers. In cases of disagreement, final values were determined after consensus discussion. The study adhered to the principles of the Declaration of Helsinki and was approved by the Showa Medical University Hospital Research Ethics Committee (Approval No. 2024–201-B). Because this was a retrospective study, instead of obtaining individual informed consent, study details were disclosed on the hospital website, providing patients with an opportunity to opt out.

### EGD and EPSIS

EGD was performed with patients in the left lateral position under sedation with 1% propofol, and CO _2_ insufflation was maintained at a flow rate of approximately 1.8 L/min using a high-flow tube (MAJ-1741; Olympus Corp., Tokyo, Japan) connected to a CO _2_ insufflator (UCR; Olympus). After completing routine EGD with a gastroscope (GIF-H290T or GIF-XZ1200; Olympus), the EPSIS procedure was initiated. For EPSIS, a disposable irrigation tube (AF-WT; Forte Grow Medical Corp., Tochigi, Japan) was connected to the working channel of a gastroscope, with the other end attached to a pressure measuring device (TR-W550, TR-TH08, AP-C35; Keyence, Osaka, Japan).

### Endoscopic observation during EPSIS

Endoscopic observation during EPSIS was conducted in a retroflex view, maintaining focus on both the cardia and the folds of the greater curvature at the same level as the cardia. When the stomach was sufficiently degassed, the grooves between the folds of the greater curvature were rarely visible. During continuous CO _2_ insufflation, the grooves between the longitudinal folds began to widen, followed by stretching of the oblique and transverse mucosal ridges between the longitudinal folds. Eventually, the mucosal ridges between the longitudinal folds flattened, and the longitudinal folds themselves became fully stretched and flattened.

**Fig. 1:**
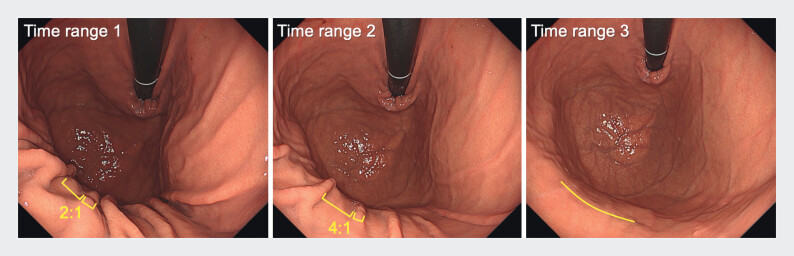
Representative image of the end of each time range.

Time ranges were defined as follows: Time range 1, until the ratio of longitudinal fold thickness to the groove width between folds reached 1:2; Time range 2, until the ratio reached 1:4; and Time range 3, until the longitudinal folds or mucosal ridges between them were almost flattened ( Fig. 1 ). IGPs were measured after complete degassing (basal IGP), at the end of each time range, and just before termination of CO _2_ insufflation (maximum IGP). CO _2_ insufflation was terminated if IGP plateaued due to belching or exceeded 20 mmHg to prevent Mallory-Weiss tears. IGPs were measured at the point of minimal respiratory variability. Following the previous report 3 , pressure difference at the end of each time range was also calculated by subtracting basal IGP from IGP at the end of each respective time range.

### Statistical analysis

Statistical analysis was performed using JMP version 18.1.0 (SAS Institute Inc., Cary, North Carolina, United States). To evaluate interobserver agreement, Cohen’s kappa statistic was used to assess concordance in determining whether the end of each time range had been reached. In cases where both observers agreed that the end of a time range had been reached, agreement for IGP values was evaluated using the intraclass correlation coefficient [ICC (2,1)]. To evaluate changes in IGP and pressure difference across the three time ranges, the Friedman test was used to assess overall differences, and Wilcoxon signed-rank tests were performed for pairwise comparisons. To assess variability of IGP measurements, the coefficient of variation was calculated by dividing the standard deviation by the mean value (SD/mean).

## Results

A total of 33 patients were enrolled in the study. Table 1 summarizes clinical characteristics and EPSIS outcomes of the enrolled patients. The most common indication for EPSIS was GERD symptoms (52%), followed by achalasia (18%). All patients presented with non-atrophic stomachs. EPSIS was terminated due to IGP exceeding 20 mmHg in 24 patients (73%), cessation of IGP increase due to belching in six patients (18%), and a flat waveform pattern in three patients (9%). No complications related to the EPSIS procedures were observed in any of the patients.

**Table:** **Table 1** Clinical characteristics of patients.

	**N = 33**
Age, mean ± SD	52.5 ± 11.9
Sex, male (%)	22 (67)
BMI, mean ± SD	23.8 ± 4.56
EPSIS results	
Basal IGP, mmHg, mean ± SD	6.4 ± 2.3
Maximum IGP, mmHg, mean ± SD	19.0 ± 3.2
Time, sec, mean ± SD	58.5 ± 20.1
Criteria of EPSIS termination (%)	
IGP exceeded 20 mmHg	24 (73)
IGP stopped increasing	9 (27)
Atrophic gastritis (%)	0 (0)
Diagnosis (%)	
GERD	17 (52)
Achalasia	6 (18)
Post anti-reflux mucosectomy	3 (9)
Others	7 (21)
BMI, body mass index; EPSIS, endoscopic pressure study integrated system; GERD, gastroesophageal reflux disease; IGP, intragastric pressure; SD, standard deviation.	

Interobserver agreement on whether the end of each time range had been reached was 0.653 for Time range 2 and 0.864 for Time range 3 ( Table 2 ). For Time range 1, all cases were judged by both observers as having reached the end of the time range ( Table 2 ). Among cases in which both observers agreed that the end of a time range had been reached, the ICC for IGPs was 0.924 for Time range 1, 0.915 for Time range 2, and 0.784 for Time range 3.

**Table:** **Table 2** Interobserver agreement.

**Time range**	**Reaching the end of time range**	**IGP at the end of time range**
Time range 1	N.A. (all reached)	0.924
Time range 2	0.653	0.915
Time range 3	0.864	0.784
IGP, intragastric pressure.		

**Fig. 2:**
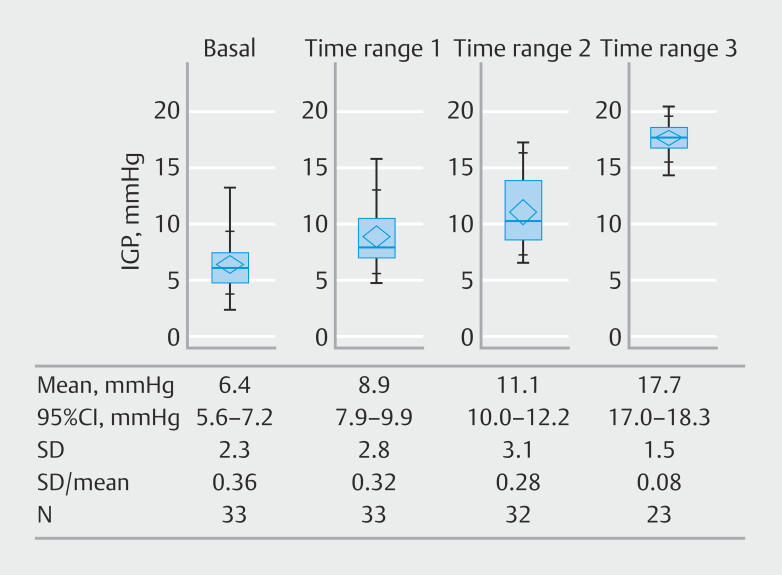
Intragastric pressure at the end of each time range. IGP, intragastric pressure; CI, confidence interval; SD, standard deviation.

Fig. 2 shows the distribution of IGP at the end of the different time ranges. Time ranges 1, 2, and 3 were fully observed in 33 (100%), 32 (97%), and 23 (70%) patients, respectively, before termination of EPSIS. Among the 10 patients in which Time range 3 was not observed, five had GERD symptoms and three had achalasia. In all three achalasia cases, EPSIS measurement was terminated because the IGP exceeded the safety threshold of 20 mmHg. Mean IGPs at the end of Time ranges 1, 2, and 3 were 8.9, 11.1, and 17.7 mmHg, respectively. Statistically significant differences were observed across the three time ranges ( *P* < 0.0001), as well as between each pairwise comparison ( *P* < 0.0001).

The coefficient of variation was lowest at the end of Time range 3 (0.08), followed by Time range 2 (0.28), and Time range 1 (0.32).

**Fig. 3:**
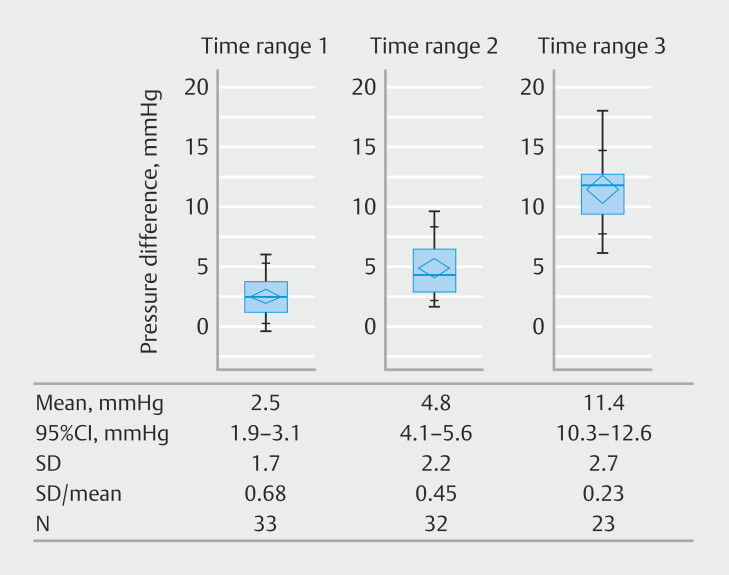
Pressure difference at the end of each time range. CI, confidence interval; SD, standard deviation.

Fig. 3 demonstrates pressure differences at the end of each time range. Mean pressure differences at the end of Time ranges 1, 2, and 3 were 2.5, 4.8, and 11.4 mmHg, respectively. Statistically significant differences were observed across the three time ranges ( *P* < 0.0001), as well as between each pairwise comparison ( *P* < 0.0001). The coefficient of variation was lowest at the end of Time range 3 (0.23), followed by Time range 2 (0.45), and Time range 1 (0.68).

**Fig. 4:**
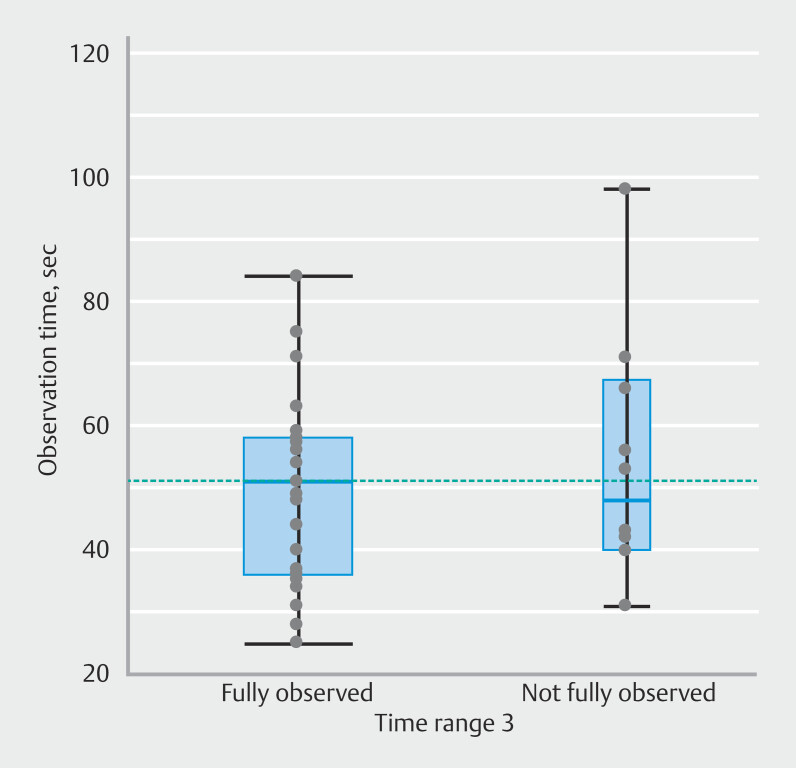
Observation time to the end of Time range 3.

Fig. 4 illustrates observation times to the end of Time range 3. For cases in which Time range 3 was not fully observed, observation time until EPSIS termination is provided. Of the 23 patients in whom Time range 3 was fully observed, mean observation time to the end of Time range 3 was 50 seconds, with a maximum of 84 seconds. In the 10 patients in whom Time range 3 was not fully observed, EPSIS was terminated in four patients when IGP had stopped increasing (range: 31–66 seconds). In the remaining six patients, the end of Time range 3 was not reached, despite the IGP exceeding 20 mmHg (mean time: 58 seconds; minimum time: 40 seconds).

## Discussion

This study demonstrated a correlation between dynamic endoscopic findings and IGP during CO _2_ insufflation using the EPSIS. Specifically, flattening of the longitudinal folds or mucosal ridges between them (the end of Time range 3) occurred at mean IGPs of 17.7 mmHg, with relatively low interindividual variation, indicating that this visual finding can serve as a reliable estimate of IGP. Moreover, assessment of the end of time range and the corresponding IGPs demonstrated high interobserver consistency, supporting reproducibility and reliability of this visual evaluation method.

Transient lower esophageal sphincter relaxation (TLESR) is known to be a significant mechanism for GERD 8 9 10 , and can be triggered by air insufflation in the stomach 11 12 . EPSIS allows for the measurement of IGP during CO _2_ insufflation, thereby enabling the assessment of LES function 1 . Insufficient elevation in IGP due to air escaping during insufflation suggests impaired LES function. Our previous findings showed that a maximum IGP < 18.7 mmHg, a flat IGP waveform, a small pressure difference between basal and maximum IGP, and a low waveform gradient were associated with GERD 1 2 3 . These results indicate that patients who achieve higher IGPs during insufflation likely have a well-functioning anti-reflux barrier. Additionally, a novel phase concept of the anti-reflux barrier with assessing IGPs has been recently proposed, incorporating not only LES function but also the gastroesophageal flap valve and esophageal peristalsis 13 . This shows the significance of estimating IGPs by dynamic endoscopic findings and assessing the functionality of LES. While pH monitoring remains the gold standard for diagnosing GERD in the absence of endoscopic findings such as Los Angeles grades C/D esophagitis or biopsy-confirmed Barrett’s esophagus, its use is limited due to the associated patient burden and prevalence of non-erosive reflux disease 14 15 . Although EPSIS is more convenient and less invasive than pH monitoring, it is not yet widely available. Estimating IGP based on real-time dynamic endoscopic findings during CO _2_ insufflation could serve as a practical alternative for evaluating LES dysfunction in clinical settings.

This study demonstrated that while widening of grooves between the longitudinal folds exhibited considerable interindividual variability, flattening of the folds or the mucosal ridges between them was more consistent across patients. Flattening of the folds or the mucosal ridges occurred in 70% of patients, with a mean IGP of 17.7 mmHg, slightly lower than the 18.7 mmHg threshold 1 , but sufficient to identify patients with suspected GERD. On the other hand, some cases did not exhibit fold or mucosal ridge flattening even at high IGPs, which could suggest a higher buffering capacity of the gastric wall against increased IGP. Factors beyond LES function such as gastric compliance, abdominal pressure, or sedation level may also contribute to the observed findings. Nevertheless, cases in which flattening of the folds or the mucosal ridges is not observed may warrant further pH monitoring, because there are instances in which IGPs fail to increase due to belching caused by LES dysfunction.

A key safety consideration is determining when to terminate continuous CO _2_ insufflation in the absence of IGP measurement using EPSIS. We used an IGP > 20 mmHg as a safety threshold to avoid complications such as Mallory-Weiss tears 1 2 3 4 5 6 7 . However, there was variability in the time taken to reach different IGP thresholds, with some patients reaching 20 mmHg in as little as 40 seconds, whereas others required up to 84 seconds for the fold or mucosal ridges between the folds to flatten. Thus, careful observation should be maintained, particularly when insufflation exceeds 40 seconds.

There were several limitations to this study. First, the retrospective design and small sample size may limit generalizability of the findings. In addition, because no specific inclusion criteria were applied regarding diagnosis or disease severity, clinical heterogeneity of the cohort may have influenced the results. Second, none of the patients in this study had atrophic gastritis, and it remains unclear whether similar results would be observed in patients with atrophic gastritis. Third, the results were obtained under sedation, which may differ in a non-sedation setting. The level of sedation may also have influenced the results. Finally, this study was not designed to evaluate the diagnostic yield of gastric fold flattening for GERD. Although we demonstrated that fold flattening was observed at a mean IGP of 17.7 mmHg, this value was not intended to serve as a diagnostic cutoff. Presence or absence of fold flattening alone cannot reliably confirm or exclude GERD, and further prospective studies are required to evaluate its diagnostic accuracy. Despite these limitations, this study demonstrated that IGP can be approximately predicted based on dynamic endoscopic findings, providing a practical approach to assessing LES function. Because EPSIS is not yet widely available, our visual, equipment-free approach may broaden access to physiological assessment during routine endoscopy.

## Conclusions

In conclusion, flattening of the gastric folds during insufflation is a reliable indicator of IGP. Based on endoscopic findings, near-complete flattening of the longitudinal folds or the mucosal ridges between them may suggest normal function, whereas a lack of flattening could indicate potential dysfunction of the anti-reflux barrier.
